# Contributions to the biodiversity of Vietnam – Results of VIETBIO inventory work and field training in Cuc Phuong National Park

**DOI:** 10.3897/BDJ.10.e77025

**Published:** 2022-01-04

**Authors:** Virginia K. Duwe, Lien Van Vu, Thomas von Rintelen, Eckhard von Raab-Straube, Stefan Schmidt, Sinh Van Nguyen, Thong Dinh Vu, Tu Van Do, Truong Hong Luu, Vuong Ba Truong, Vanessa Di Vincenzo, Olga Schmidt, Falko Glöckler, Regine Jahn, Robert Lücking, Katharina C. M. von Oheimb, Parm Viktor von Oheimb, Sandra Heinze, Nelida Abarca, Sarah Bollendorff, Thomas Borsch, Eliana Buenaventura, Huong Thi Thu Dang, Thuy Dieu Dinh, Hai Thi Do, Sarah Ehlers, Jörg Freyhof, Sofía Hayden, Peter Hein, Tuan Anh Hoang, Duc Minh Hoang, Son Nghia Hoang, Harald Kürschner, Wolf-Henning Kusber, Han Ngoc Le, Trang Quynh Le, Mattes Linde, Wolfram Mey, Hiep Duc Nguyen, Man Thi Nguyen, Minh Trung Nguyen, Dat Van Nguyen, Tu Van Nguyen, Vu Dang Hoang Nguyen, Dat Quoc Nguyen, Michael Ohl, Gerald Parolly, Tan Nhat Pham, Phu Van Pham, Katharina Rabe, Bernhard Schurian, Oliver Skibbe, Anna Sulikowska-Drozd, Quang Van To, Tam Quang Truong, Jonas Zimmermann, Christoph L. Häuser

**Affiliations:** 1 Museum für Naturkunde Berlin – Leibniz Institute for Evolution and Biodiversity Science, Berlin, Germany Museum für Naturkunde Berlin – Leibniz Institute for Evolution and Biodiversity Science Berlin Germany; 2 Vietnam National Museum of Nature, Vietnam Academy of Science and Technology, Hanoi, Vietnam Vietnam National Museum of Nature, Vietnam Academy of Science and Technology Hanoi Vietnam; 3 Botanic Garden and Botanical Museum, Freie Universität Berlin, Berlin, Germany Botanic Garden and Botanical Museum, Freie Universität Berlin Berlin Germany; 4 SNSB-Zoologische Staatssammlung München, Munich, Germany SNSB-Zoologische Staatssammlung München Munich Germany; 5 Institute of Ecology and Biological Resources, Vietnam Academy of Science and Technology, Hanoi, Vietnam Institute of Ecology and Biological Resources, Vietnam Academy of Science and Technology Hanoi Vietnam; 6 Graduated University of Science and Technology, Hanoi, Vietnam Graduated University of Science and Technology Hanoi Vietnam; 7 Southern Institute of Ecology, Vietnam Academy of Science and Technology, Ho Chi Minh City, Vietnam Southern Institute of Ecology, Vietnam Academy of Science and Technology Ho Chi Minh City Vietnam; 8 Institute of Tropical Biology, Vietnam Academy of Science and Technology, Ho Chi Minh City, Vietnam Institute of Tropical Biology, Vietnam Academy of Science and Technology Ho Chi Minh City Vietnam; 9 Freie Universität Berlin, Institut für Biologie, Systematische Botanik und Pflanzengeographie, Berlin, Germany Freie Universität Berlin, Institut für Biologie, Systematische Botanik und Pflanzengeographie Berlin Germany; 10 Universität Greifswald, Greifswald, Germany Universität Greifswald Greifswald Germany; 11 Vietnam Forest Museum, Hanoi, Vietnam Vietnam Forest Museum Hanoi Vietnam; 12 University of Lodz, Lodz, Poland University of Lodz Lodz Poland

**Keywords:** VIETBIO, Vietnam, biodiversity discovery, species inventory, Cuc Phuong National Park, MyFieldBook app

## Abstract

VIETBIO [Innovative approaches to biodiversity discovery and characterisation in Vietnam] is a bilateral German-Vietnamese research and capacity building project focusing on the development and transfer of new methods and technology towards an integrated biodiversity discovery and monitoring system for Vietnam. Dedicated field training and testing of innovative methodologies were undertaken in Cuc Phuong National Park as part and with support of the project, which led to the new biodiversity data and records made available in this article collection.

VIETBIO is a collaboration between the Museum für Naturkunde Berlin – Leibniz Institute for Evolution and Biodiversity Science (MfN), the Botanic Garden and Botanical Museum, Freie Universität Berlin (BGBM) and the Vietnam National Museum of Nature (VNMN), the Institute of Ecology and Biological Resources (IEBR), the Southern Institute of Ecology (SIE), as well as the Institute of Tropical Biology (ITB); all Vietnamese institutions belong to the Vietnam Academy of Science and Technology (VAST).

The article collection "VIETBIO" (https://doi.org/10.3897/bdj.coll.63) reports original results of recent biodiversity recording and survey work undertaken in Cuc Phuong National Park, northern Vietnam, under the framework of the VIETBIO project. The collection consist of this “main” cover paper – characterising the study area, the general project approaches and activities, while also giving an extensive overview on previous studies from this area – followed by individual papers for higher taxa as studied during the project. The main purpose is to make primary biodiversity records openly available, including several new and interesting findings for this biodiversity-rich conservation area. All individual data papers with their respective primary records are expected to provide useful baselines for further taxonomic, phylogenetic, ecological and conservation-related studies on the respective taxa and, thus, will be maintained as separate datasets, including separate GUIDs also for further updating.

## Introduction

Vietnam is part of the Indo-Burma biodiversity hotspot, one of the 25 hotspots of this kind in the world (Myers et al. 2000). The country harbours a rich biodiversity and high levels of regional and local endemism, ranking 25^th^ worldwide in plant, bird and mammal species diversity per unit area (Groombridge and Jenkins 2002). Vascular plant diversity is estimated with 12,000 species with high levels of endemism around 30% countrywide and perhaps reaching 50% in northern Vietnam (Regalado et al. 2005). The endemism of fauna in Vietnam is the highest in Indochina (Vietnam, Laos, Cambodia) (MacKinnon 1997, Sterling and Hurley 2005). Vietnam harbours some of Asia's most endangered animals, such as the Crested Argus (*Rheinardiaocellata*), Edwards Pheasant (*Lophuraedwardsi*), Green Peafowl (*Pavomuticus*), the Doucs (*Pygathrix* spp.), Delacour Langur (*Trachypithecusdelacouri*) and Tonkin Sub-nosed Monkey (*Rhinopithecusavunculus*) (Rambaldi et al. 2001).

Threats to Vietnam's biodiversity are habitat fragmentation and, above all, the degradation of forest vegetation due to logging, agricultural conversion, as well as wild fires. Massive over-utilisation due to overgrazing, hunting or collecting rare medicinal plants and timbers has a major impact on the decline of biodiversity as well (MacKinnon 1997, Rambaldi et al. 2001, Ngo et al. 2020). Protected areas such as nature reserves, national parks or landscape conservation areas play an increasingly important part in the conservation and protection of Vietnam's rich and endemic biodiversity. Cuc Phuong National Park was founded as the first national park in northern Vietnam in 1962 (Sterling et al. 2006). In the following decades, many more protected areas were established. As a result, currently there is a large number of national parks and other protected areas (126 forest protected areas, 68 wetland protected areas, 15 marine protected areas) in the country (The Socialist Republic of Vietnam 2003).

Protected areas are essential for biodiversity conservation. Globally, species richness and abundance have often been shown to be higher in protected areas, particularly due to differences in land use between protected and unprotected habitats (Gray et al. 2016). Therefore, the establishment and extension of ecologically representative and well-connected protected areas is also a priority under the Convention on Biological Diversity (Aichi Biodiversity Target 11; CBD 2010). However, in order to manage protected areas and conserve and sustainably use biodiversity in general, we need to know biodiversity beyond a few keystone species and must be able to monitor it. An inventory of the enormous diversity of smaller organisms, especially invertebrates, algae and fungi and their interactions, is, therefore, a requirement and necessary as a benchmark to effectively monitor the maintenance of biodiversity under specific conservation and land management regimes and, thus, for the long-term conservation of biodiversity. However, vascular plants as major constituents and primary producers in terrestral ecosystems also need to be known at species level.

In addition to a comprehensive inventory of species, tools to easily identify these species are of practical relevance for a sound and sustainable biodiversity management of any protected area.This paper attempts to provide some primary data from recent fieldwork in the context of the bilateral German-Vietnamese VIETBIO project towards a more comprehensive species inventory of Cuc Phuong National Park in northern Vietnam. This editorial provides a general introduction and project description, as well as an overview of the approach and methods applied and will be supplemented by individual papers on particular taxa, including all new primary data generated.

## The VIETBIO project: Innovative Approaches to Biodiversity Discovery and Characterisation in Vietnam

VIETBIO is a German-Vietnamese training and research project, focusing on the development and transfer of an integrated biodiversity discovery and monitoring system for Vietnam. The project aims to strengthen the capacities for research on the conservation and innovative and sustainable use of biodiversity in Vietnam and beyond in Indochina through the installation of a national capacity network.

The project is implemented through a collaboration between the Museum für Naturkunde Berlin - Leibniz Institute for Evolution and Biodiversity Science (**MfN**), the Botanic Garden and Botanical Museum, Freie Universität Berlin (**BGBM**) and four Vietnamese institutes nationally leading in collection-based biodiversity research: The Vietnam National Museum of Nature (**VNMN**) and the Institute of Ecology and Biological Resources (**IEBR**) in Hanoi, as well as the Southern Institute of Ecology (**SIE**) and the Institute of Tropical Biology (**ITB**) in Ho Chi Minh City. All four belong to the Vietnam Academy of Science and Technology (**VAST**).

**Figure 1.**
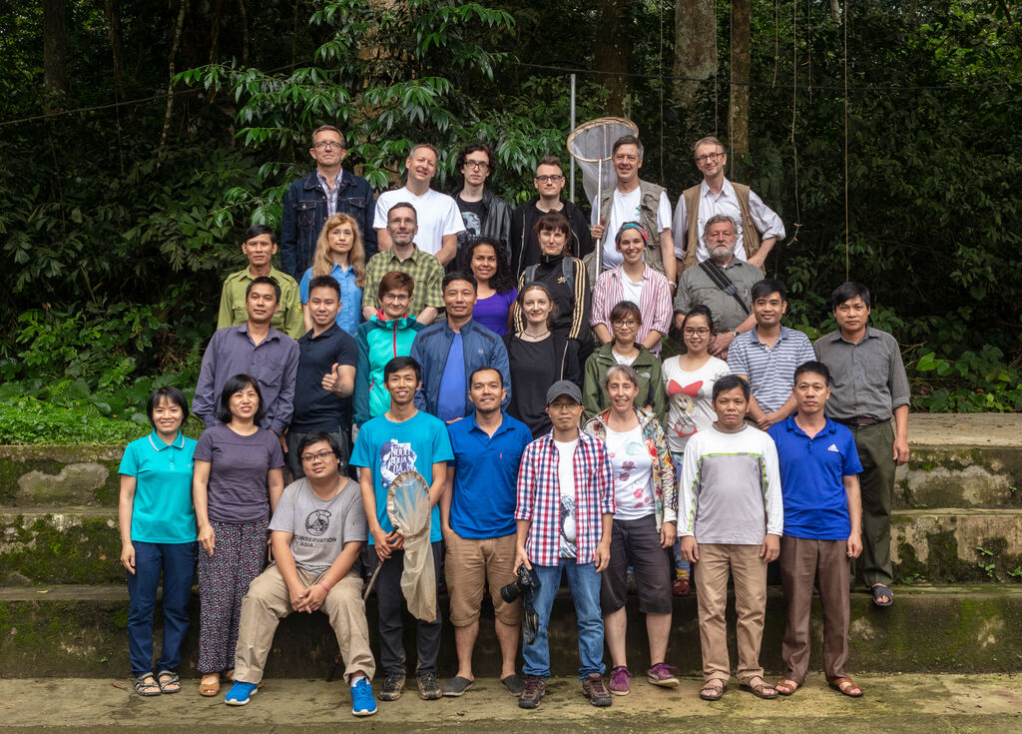
Participants of the VIETBIO training workshop at Cuc Phuong National Park in Vietnam (May 2019).

The VIETBIO project (2017-2021) is a flagship initiative for the implementation of the 'Action and Research Plan on Biodiversity' that was issued by the German Federal Ministry of Education and Research (BMBF). This governmental initiative aims at addressing global challenges in biodiversity conservation and the development of viable solutions through research and innovation. The project entails two basic activities: firstly, joint field sessions in Vietnam to collect new data and to obtain samples/specimens for training purposes and for testing innovative inventory and recording methods and tools in the field (Fig. 1); secondly, training of Vietnamese researchers and technicians in state-of-the-art methods during working visits of three months to Germany (MfN and BGBM Berlin), which are organised in four modules: specimen digitisation, DNA barcoding, data management and bioacoustics. State-of-the-art equipment purchased for the training is being used to document and analyse the Vietnamese samples/specimens collected in the field and will subsequently be transferred to the Vietnamese partners for its continued application at their institutions.

In this way, VIETBIO combines the application of modern technologies with the training of biodiversity scientists in the species-rich tropical country in order to establish and strengthen independent biodiversity research in Vietnam within international networks.

## The study area: Cuc Phuong National Park

The recordings and studies were undertaken during a field trip to Cuc Phuong National Park from 29 April until 10 May 2019. The field campaign was undertaken by the following participants:

Christoph L. Häuser, Thomas von Rintelen, Sarah Ehlers, Michael Ohl, Sofia Hayden, Bernhard Schurian, Katharina C. M. von Oheimb, Parm Viktor von Oheimb, Wolfram Mey (MfN); Mattes Linde (Universität Greifswald); Sarah Bollendorff, Eckhard von Raab-Straube (BGBM); Anna Sulikowska-Drozd (University of Lodz); Olga Schmidt, Stefan Schmidt (SNSB-Zoologische Staatssammlung München); Lien Van Vu, Trang Quynh Le, Tuan Anh Hoang (VNMN); Vu Dang Hoang Nguyen, Vuong Ba Truong (ITB); Hiep Duc Nguyen, Han Ngoc Le, Huong Thi Thu Dang, Phu Van Pham, Tu Van Do, Thong Ding Vu (IEBR); Quang Van To, Dat Quoc Nguyen (SIE); Thuy Dieu Dinh (Hanoi National University of Education) and Tan Nhat Pham (Vietnam Forest Museum).

### Location and topography

**Figure 2.**
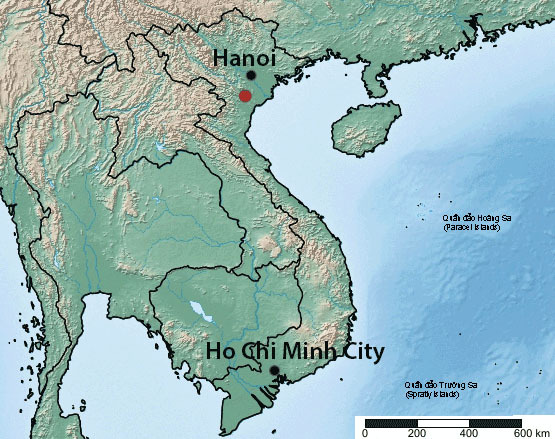
Location of Cuc Phuong National Park in Vietnam.

Cuc Phuong National Park is located about 120 km SW of the capital Hanoi along the southern edge of the Red River (Song Hong) Delta, between 20°14′ to 20°24’ N and 105°29' to 105°44' E (Fig. 2). The Park covers an area of 22,200 ha, with a length of about 30 km and a width of about 6 to 10 km. Cuc Phuong National Park extends across three Provinces (Ninh Binh: 11,350 ha, 2 communes, 51.1% of total area; Hoa Binh: 5,850 ha, 9 communes, 26.4% of total area; Thanh Hoa: 5,000 ha, 3 communes, 22.5% of total area) and is divided into a Strict Protection Zone (16,800 ha), an Ecological Rehabilitation Zone (3,600 ha), an Administration and Service Zone (1,800 ha) and a Buffer Zone (Cuc Phuong National Park 2010). In addition, the Park is surrounded by a buffer zone that extends beyond the Park boundaries (Rugendyke and Son 2005).

The topography is characterised by limestone karst and the Park extends along the foothills of a limestone mountain range running from northwest to southeast, including a central, broad valley with an average elevation of 400-450 m above sea level (a.s.l.). The highest point of the Park, May Bac peak at 648 m a.s.l., is located in its north-western corner (Cuc Phuong National Park 2012). The underlying geological formations for the region are mostly sedimentary rocks from the early Mesozoic (Middle Triassic) and the geomorphology of the Cuc Phuong area is shaped by a special type of semi-covered karst, with typical karst outcrops and formations where these reach the surface, such as karren karst, sink-holes and numerous karstic (also underground) caves. Seven different soil types have been recorded, which belong to two main types, depending on whether they are derived from underlying limestone sediments or other, mostly metamorphic bedrock. The hydrology is also typical for a karst-dominated landscape and besides the Buoi and Ngang Rivers in the western part of the Park, there are many dry streams only appearing in the rainy season, many caves and underground flows and little permanent surface water (Cuc Phuong National Park 2010).

### Weather and climate

For the Cuc Phuong National Park, the annual average temperature is 22.5°C, with a maximum and minimum annual average of 32.2°C and 15.8°C, respectively. The lowest monthly average temperature recorded was 5.3°C (January 1993) and the highest monthly temperature average was 38.4°C (June 1997). Air humidity is generally high, with an annual average humidity of 84.8%. Relative humidity is usually highest in the first months of the year (January-April) and lowest in the last months of the year (October-December) (Truong et al. 2018).

Average rainfall for Cuc Phuong National Park is about 1,680 mm/year, with a recorded minimum of 1,126 mm/year (1998) and a maximum of 2,194 mm/year (1996). Most rainfall occurs between May and October (89.1% of the annual average rainfall). In combination with the regional temperature regime, this accounts for two distinct seasons over the year: a hot rainy season from May to October with an average temperature of 26.4°C and a dry and cooler season from November to April with an average temperature of 18.6°C. During the hot rainy season a south-eastern monsoon prevails, with average wind speeds of 4-12 m/s. In the dry and cooler season, there is a north-eastern monsoon with wind speeds of 4-20 m/s, usually combined with dry cold air and some drizzle at the end of the season (Truong et al. 2018). As well as other areas in the greater vicinity of the Gulf of Tonkin, Cuc Phuong National Park is regularly affected by tropical depressions and storms, often with heavy winds and rainfall causing tree falls and other damage to the forest and landscape.

### Biodiversity

Cuc Phuong National Park is one of the areas with the highest documented biological diversity in Vietnam and has attracted many researchers and naturalists leading to a comparatively well-studied flora and (mostly vertebrate) fauna. Surveys and studies undertaken have confirmed that, for several animal and plant groups, Cuc Phuong National Park harbours more than half of the total number of higher taxa and, in some groups, more than one third of the total species, recorded for Vietnam (e.g. Nguyen 1992, Larsen et al. 2005, Eguchi et al. 2011, Cuc Phuong National Park 2012, Truong et al. 2018). A total of 20,526 records (plants, animals, fungi) are currently available online via GBIF (GBIF.org 2021a) for Cuc Phuong National Park and the surrounding area. Of these, 16,323 are based on "human observations" and 3,796 on "preserved specimens". The high biodiversity of Cuc Phuong National Park results not only from the area’s varied landscape and great diversity in microhabitats, but also from its geographic location at the interface between temperate subtropical and tropical, monsoon-dominated climates and different biogeographic regions.

Here, we present only some brief and general information about flora, algae, funga and fauna and further details about the individual taxa surveyed during the VIETBIO project are included in the respective data papers to follow this introduction.

### Flora

The main vegetation cover of Cuc Phuong National Park is broad-leaved tropical evergreen lowland forest, with considerable areas of primary forest found mainly along the limestone mountain ridges and in the valleys of the Park centre. Differences in soil layer lead to different forest canopies, which are generally taller and denser in the valleys than on slopes, ridges and peaks (Soejarto et al. 2004). Averyanov et al. (2013) determined two main forest variants: forests on rocky limestone and forests of flat alluvial valleys. Based on the types of topography and soils, Nguyen (1997) further divided the plant diversity into four communities: plants on the summits of limestone mountains (including 65 common species, such as *Quercus* sp., *Jasminumlanceolarium* and *Dendrobiumdentatum*), plants on slopes of limestone mountains (about 200 species belonging to the Pteridophytes, Gymnospermae and Angiospermae), plants in valleys (1,219 species belonging to the Bryophyta, Pteridophyta and Angiospermae) and plants on non-limestone soil (440 species, especially from the families Lauraceae, Meliaceae, Fagaceae, Elaeocarpaceae and Euphorbiaceae). He also noted a decreasing number of plant species from the valley to the top of the mountains.

Being one of Vietnam’s seven international centres of plant diversity (Davis et al. 1995), Cuc Phuong National Park contains flora which is amongst the best known and documented in Vietnam. While botanical work started early and plant records from Cuc Phuong National Park have been mentioned in several works (e.g. Lecomte and Gagnepain 1950, Pham 1993, Pham 1999, Middleton 2014, Hul and Dy Phon 2014, Staples 2018), the Park’s first list of plants was published in 1971 by the Cuc Phuong Sub-Institute of Forestry (1971). It was updated by Nguyen (1992), who listed 1,942 species, including 126 bryophytes, recorded by Tran (1992). Phung et al. (1996) published a new list of 1,944 plants including 127 bryophytes, one psilotophyte, nine lycopodiophytes, one equisetophyte, 127 polypodiophytes, three gymnosperms and 1,676 angiosperms. Le et al. (1997) reported another list of 1,983 species, including 126 bryophytes, one psilotophyte, nine lycopodiophytes, one equisetophyte, 129 polypodiophytes, five gymnosperms and 1,712 angiosperms. The latter two publications were mainly based on Nguyen (1992) with additional records. In addition, Nguyen (1997) catalogued 1,658 flowering plants for the Park. Nguyen et al. (2001) and Nguyen et al. (2002) highlighted the Plant inventory work at Cuc Phuong National Park undertaken during 1991-2000.

The most recent update of the seed plant flora was done by Soejarto et al. (2004). They revised all the previous lists, re-identified collections deposited in the Herbarium of Cuc Phuong National Park since the 1960s and added their own new collections from 1998-2003 to the Park's Herbarium. They came up with a total number of 1,926 seed plant species (gymnosperms and angiosperms), with a corresponding list available online at http://fm2.fieldmuseum.org/plantatlas/about.asp (Soejarto et al. 2001). According to the authors, however, this number does not reflect the full diversity of plants in the Park, as specimens deposited at other national and international herbaria have not been re-examined and further new species for the Park are awaiting discovery. Indeed, new records from the Park are still being published, for example, Rehse and Kress (2003), van Sam and Nooteboom (2007), Averyanov et al. (2016) and Tran et al. (2020). Based on the work of Soejarto et al. (2004), a guidebook of 294 common plants of the Park was published (Nguyen et al. 2009). Furthermore, identification manuals for selected plant groups have been prepared in recent years (Soejarto and Phan 2005, Averyanov and Averyanov 2006, Le 2008, Nguyen 2009, Averyanov et al. 2013). Through the Global Biodiversity Information Facility (GBIF), 3,228 plant records (22 human observations and 3,114 preserved specimens) are currently available for Cuc Phuong National Park and the surrounding area. Most data correspond to Tracheophyta (3,020 specimens) and few to Bryophyta (180 specimens). Within Tracheophyta, most records are from dicotyledons (class Magnoliopsida in GBIF) with 2,527 preserved specimens and monocotyledons (class Liliopsida in GBIF) with 414 preserved specimens (GBIF.org 2021a). These numbers exclude the 219 plant specimens of the Herbarium Berolinense B, collected during the current field campaign in 2019.

Amongst the reported plants are 118 species threatened according to the current IUCN Red List (IUCN 2021) and Vietnam’s Red Data Book (Nguyen et al. 2007), 11 species are endemic to the Park, 433 species have medicinal value, 229 species are edible and 240 species can provide dyes.

There is a 167 ha botanic garden located in the Park, which harbours, for ex situ conservation, 811 valuable plant species, including 210 trees native to the Park, 85 trees from other parts of Vietnam, five exotics, 25 aroids native to the Park, 20 fruity plants, 15 bamboos, 17 cycads, 15 palms, 296 medicinal plants and 140 orchids.

### Algae

Most occurrences for algae in Vietnam have been documented for marine algae because of the country´s long coastline, for example, for Ochrophyta (GBIF.org 2021b). Neither checklists of freshwater algae for the country nor comprehensive datasets for specific regions or algae groups are currently available. Records are very patchy and new species were often published as individual "exotics" within European diatom floras (e.g. Hustedt 1966, Levkov et al. 2014) or within studies on specific groups from the South East Asian Region (e.g. Reichardt 1997, Reichardt 2005). Until 2014, only three diatom taxa were described from Vietnam. For the overall region of South East Asia, 19 species were described as new, 17 of them being diatoms (Jahn and Kusber 2005, continuously updated).

In the last decade, however, more than 25 papers on freshwater microalgae were published, with the description of many new taxa in the diatoms (bacillariophytes) (e.g. Glushchenko et al. 2019, Glushchenko et al. 2020, Kezlya et al. 2020a, Kezlya et al. 2020b, Kulikovskiy et al. 2020a, Kulikovskiy et al. 2020b, Kulikovskiy et al. 2021) and chrysophytes (e.g. Gusev et al. 2021) from Vietnam (PhycoBank 2021). The focus of these publications was the southern part of Vietnam and especially on Cat Tien National Park. However, so far no research has been done on freshwater diatoms of Cuc Phuong National Park.

### Funga

The Vietnamese funga (including lichen-formers) are only moderately known, with relatively few and scattered studies. A first checklist of Vietnamese macrofungi (Kiet 1998) listed 829 species, while Mel'Nik et al. (2016) reported 57 species of anamorphic (asexually reproducing) microfungi. Duong et al. (2011) listed 40 species of *Xylaria* for the country. The most recent checklist of lichenised fungi (Aptroot and Sparrius 2006) enumerated 275 taxa, a number that increased to nearly 500 in the online checklist by Gueidan (2013). In addition, 57 species of myxomycetes have been reported (Tran et al. 2014). Including other reports and taxonomic works (e.g. Dörfelt et al. 2004, Nguyen et al. 2011, Schumm and Aptroot 2012, Gueidan et al. 2014, Joshi et al. 2018, Pham and Morozova 2020, Zhurbenko et al. 2020), well over 1,500 species of fungi and fungal-like organisms are known from Vietnam, still a relatively low number considering that similar-sized, largely temperate countries in the Northern Hemisphere, whose funga are relatively well studied, report 23,000 (Italy), 18,000 (UK), 15,000 (Germany) and 14,000 (Japan) species, respectively (Gaya et al. 2021).

There are few comprehensive listings for the funga of Cuc Phuong National Park. The main work is an illustrated guide to 214 species of macrofungi (Tran and Truong 2005). Thirty species of Psathyrellaceae were reported by Le and Doan (2018) and nine species of *Xylaria* by Duong et al. (2011). A checklist of myxomycetes encompasses 25 species (Tran et al. 2014). A GBIF dataset, compiled by Luong et al. (2020), included ten lichenised species, collected by Tamás Pócs and housed at Herbarium Eger in Hungary (EGR), only three being identified to species level. Some novel basidiomycete mushrooms were reported by Le and Chu (2018) (*Volvariella*), Büttner et al. (2020a), Büttner et al. (2020b) (*Candolleomyces, Psathyrella*) and Lam and Hao (2013) provided records of *Biscogniauxia* (Xylariaceae). As part of the present project, Lücking et al. (2020) provided a detailed study on the feasibility of DNA barcoding of fungi, identifying the polypore *Cubamycesmenziesii* for the Park. Some scattered works dealt with microfungi in diverse groups (hyphomycetes: Yen et al. 2020; yeasts: Luong et al. 2000, Luong et al. 2005). In a preliminary study on fungi isolated from soil samples, Yen et al. (2008) reported 256 cultures corresponding to 51 genera of microfungi. Many studies on Vietnamese fungi have focused on biochemistry and biotechnology, including several based on material of macrofungi (*Phanerochaetechrysosporium*, *Trametesmaxima*, *Trametesversicolor*, *Xylariaschweinitzii*) and microfungi (*Fusariumproliferatum*, *Trichodermareesei*) from Cuc Phuong National Park (Yen et al. 2010, Gao et al. 2012, Lam and Chien 2013, Linh et al. 2014, Giap and Hiep 2020). Overall, more than 300 species of fungi including some lichen-formers have been reported from the Park.

### Fauna

The vertebrate fauna of Cuc Phuong National Park is comparatively well known and systematic studies have been carried out since 1963. Until recently, more than 660 vertebrate species from 35 orders and 120 families have been recorded for the Park, of which 73 species are listed in the Vietnam Red List (Ministry of Science and Technology – Vietnam Academy of Science and Technology 2007). In particular, 136 species of mammals (accounting for nearly 50% of all mammals of Vietnam), 336 species of birds (39% of the total bird species of Vietnam), 78 species of reptiles, 46 species of amphibians and 66 species of fishes (10% of freshwater fishes of Vietnam) have been recorded for Cuc Phuong National Park (Cuc Phuong National Park 2012). These include a number of very rare taxa, some of them potential endemics, which were discovered in Cuc Phuong National Park, such as the ferret badger *Melogalecucphuongensis*, recently also found in southern China (Li et al. 2019), the cave dwelling gecko *Cyrtodactyluscucphuongensis* and the silurid catfish *Pterocryptiscucphuongensis*. Through GBIF, 15,754 records of Chordata are currently available from Cuc Phuong National Park. With 15,614 records, almost all of them human observations, the majority of these data are from birds, followed by just 91 records from reptiles (GBIF.org 2021a, data download Cuc Phuong NP and the surrounding area, 2021_04). While the species inventory for mammals and birds can be considered as fairly complete (see Feiler et al. 2008), for other vertebrate groups, new records and even species new to science have recently been reported in the Park (e.g. Nguyen et al. 2018, Poyarkov et al. 2018) and continuous monitoring data for many vertebrate groups are much needed.

The knowledge on the invertebrate fauna of Cuc Phuong National Park is relatively limited and only a few groups have been studied in more detail, such as ants (Yamane et al. 2003), butterflies (Ikeda et al. 1998, Ikeda et al. 1999, Ikeda et al. 2000, Ikeda et al. 2001a, Ikeda et al. 2001b, Ikeda et al. 2002, Luong et al. 2004), cicadas (Do and Nguyen 2016), dragonflies (Do et al. 2011), some larger beetles (Wiesner et al. 2017), millipedes (Nguyen et al. 2019a, Nguyen et al. 2019b), some spiders (Ono et al. 2012), freshwater crustacea (Do et al. 2016) and molluscs (Vermeulen and Whitten 1998, Vermeulen and Maassen 2003). According to the 2010 species list for Cuc Phuong National Park (Cuc Phuong National Park 2010) and additional sources (Cuc Phuong National Park 2010, Nguyen et al. 2012, Nguyen et al. 2014, Do et al. 2016, Wiesner et al. 2017), there are 2,030 invertebrate species recorded for the Park, of which more than 1,770 are insects, 29 crustaceans, 19 myriapods, 29 arachnids, 52 annelids, 129 molluscs and six species are nematodes. Through GBIF, currently only 1,071 records are available for Arthropoda from Cuc Phuong National Park, most of them for Insecta (923 records in total, with Lepidoptera: 447, Odonata: 181 and Hymenoptera: 68 records; GBIF.org 2021a, data download Cuc Phuong National Park and the surrounding area, 2021_04). The 363 records available for Mollusca are almost entirely from terrestrial gastropods (GBIF.org 2021a, data download Cuc Phuong NP and surrounding area, 2021_04). The terrestrial gastropod fauna of Vietnam is highly diverse and over 850 species have been described from the country (Raheem et al. 2017). These molluscs particularly flourish in calcium-rich limestone karst habitats (Clements et al. 2006), such as those in Cuc Phuong National Park, while different karst areas in the same overall region have been found to differ considerably from each other in species composition (Vermeulen and Maassen 2003, von Oheimb et al. 2018). Overall, invertebrate species numbers for Cuc Phuong National Park are likely substantially underestimated, even for the better studied groups. In addition, several inventories list a number of taxa not identified to species or even genus level and many species recorded have not been reliably identified. Therefore, many invertebrate checklists for the Park still have to be considered as preliminary and the lack of taxonomic expertise and resources remains a major barrier towards quick progress.

Still, striking new insect and other invertebrate species are continuously being discovered and described from Cuc Phuong National Park (e.g. Hennemann and Conle 1997, Schintlmeister 1997, Arita and Gorbunov 2000, Byun and Park 2007, Lin et al. 2009, Cook et al. 2010, Long and van Achterberg 2015, Xin et al. 2015, Ivanova et al. 2016, Fabrizi et al. 2019, von Oheimb et al. 2019) and also during the VIETBIO fieldwork, numerous new records for invertebrates were made and will be published and added to the inventory. The goal of a comprehensive inventory for many invertebrate animal groups of Cuc Phuong National Park is still far from being completed and will require further substantive attention and support.

### Conservation

Founded nearly 60 years ago, Cuc Phuong National Park has become an international hub for several dedicated long-term conservation projects. The Endangered Primate Rescue Center (EPRC) was founded in 1993 through a collaboration between the Frankfurt Zoological Society and Cuc Phuong National Park, which was joined, in 2013, by the Leipzig Zoo. With the goal to conserve endangered primate species through rescue and breeding programmes, more than 180 animals have been raised at the Center, some being the first of their species to be born in captivity, including the critically-endangered Cat Ba Langur (*Trachypithecuspoliocephalus*), Delacour Langur (*Trachypithecusdelacouri*) and the Grey-Shanked Douc Langur (*Pygathrixcinerea*). Successful releases of captivity-bred animals started in 2007 and have taken place at a number of protected areas across Vietnam. Today, the Center cares for around 180 animals representing 15 species and is a major attraction for visitors to the Park (https://www.eprc.asia).

As another permanent installation, the Turtle Conservation Center (TCC) was founded in 1998 by Fauna and Flora International (FFI) as part of a larger conservation initiative focused on Cuc Phuong National Park. In 2001, management of the project was transferred to Cuc Phuong National Park and, today, the TCC is seen as a flagship for efforts to protect tortoises and freshwater turtles in Vietnam and in the South East Asian region (https://asianturtlenetwork.org/project%20profiles/vietnam/cuc_phuong.htm). This is further complemented by a special Carnivore and Pangolin Conservation Programme (CPCP) established in 2005, which is operated jointly by Cuc Phung National Park and Save Vietnam's Wildlife (https://www.svw.vn). Both Centers and offices of these programmes are located near the main entrance to the Park and help to attract increasing numbers of visitors and tourists.

## Approach and methods

**Figure 3.**
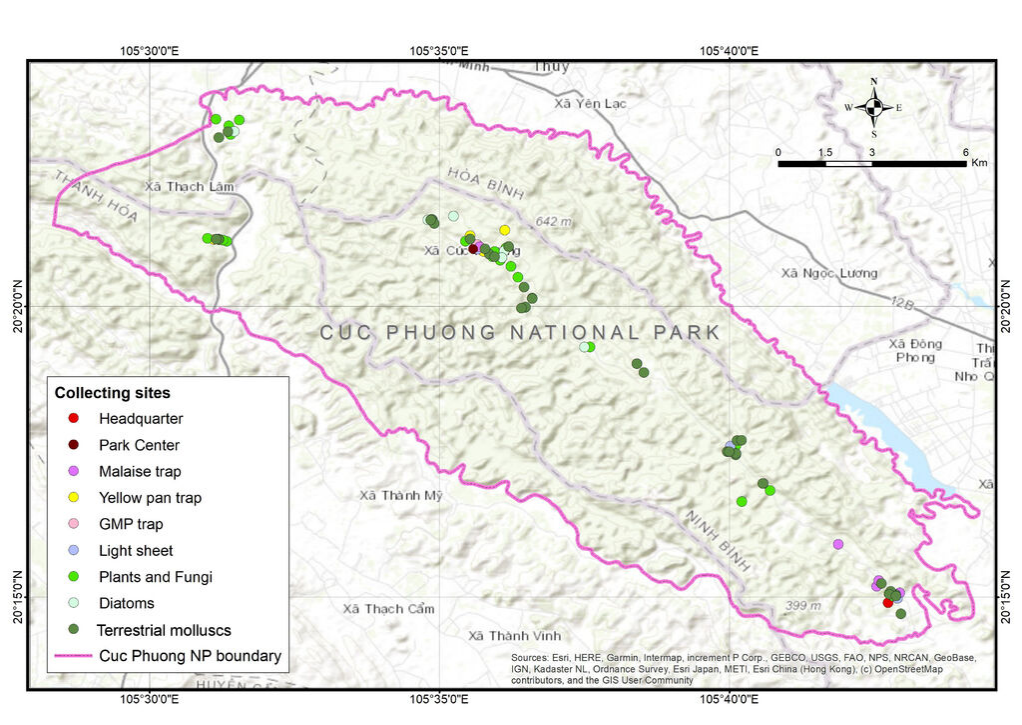
Recording sites in Cuc Phuong National Park during the 2019 field training course.

During short visits by individual members between May 2018 and end of 2019 and a main field trip with the whole team from 29 April until 10 May 2019, samples were taken at different sites in Cuc Phuong National Park (Fig. 3).

The study focused on several taxonomic groups spanning the terrestrial and aquatic fauna, as well as the terrestrial flora, funga (including lichens) and aquatic diatoms of Cuc Phuong National Park. The main purpose was to record and to provide detailed primary occurrence data of individual species for the Park, with the aim to record as many different species as possible for the groups surveyed during the study.

### Field recording and collecting methods

For data recording and sampling in the field, the following methods and protocols were applied:

#### Terrestrial fauna

**Malaise traps**

Malaise traps (Malaise 1937) are efficient tools for collecting flying insects. They are often employed in biodiversity and monitoring surveys because they allow extensive and dense sampling as part of long-term studies (Hebert et al. 2016, Karlsson et al. 2020). The specimens collected and preserved in 80-95% ethanol are most suitable for obtaining DNA sequence data. The specimens from ethanol samples, for example, insects such as Lepidoptera, can also be used for morphological analysis (Schmidt et al. 2019).

During the course of the field study, five Malaise traps were operated for 2-5 days across a range of different habitats between 160 m and 390 m a.s.l. in Cuc Phuong National Park (Fig. 3). An additional trap was set up near the Park centre (Fig. 3) as part of the Global Malaise Programme (GMP; http://biodiversitygenomics.net/projects/gmp/), which was operated for a whole year, starting in May 2019, with bottles changed twice a week.

**Yellow pan traps**

Yellow pan traps (YPT) are yellow-coloured dishes or bowls that are partially filled with water (or ethanol) with a small amount of preferably unscented detergent to reduce surface tension. YPTs catch insects that are attracted to the yellow colour of the dish, in particular flower-visiting insects, including many Diptera and Hymenoptera. YPTs allow to sample a wide range of different microhabitats and they are economic, easy to set up and can be placed in almost any location (Masner 2009). Each day during the field trip, about 50 YPTs were placed along a transect, about 5-10 m apart from each other. The traps were operated for 4-8 hours and insects transferred to 80% ethanol.

**Hand collecting**

Flying insects, especially butterflies (Papilionoidea), Hymenoptera and Odonata, were collected by hand with standard butterfly nets, usually with long handles. For insects with low abundances or such that occur clumped at few locations, including beetles and sawflies, a sweep net was used as the primary collecting device. In addition, hand collecting was employed when appropriate, for example, for collecting larval stages of sawflies or for selectively picking moths and other insects from light sheets and light towers or from other surfaces. Live specimens and empty shells of terrestrial molluscs were detected by visual search, a standard method for species inventory in this group (Cameron and Pokryszko 2005) and collected by hand, partly using forceps.

**Collecting at lights (UV and mercury vapour lamps)**

At night, Lepidoptera and other nocturnal insects were collected by attracting them with different types of light sources: (1) a mercury vapour light bulb (125 W) running from a generator, (2) a light emitting diode (LED) lamp (entoLED, bioform entomology) running from rechargeable 12 V batteries and (3) a UV light set including a black light and a fluorescent light tube (8 W each) (see also Schmidt et al. 2017). The light sources were either placed inside a standard gauze tower construction approximately 2 m high or in front of a 200 x 300 cm white linen sheet and, if required, protected from the rain by an umbrella. In addition, specimens attracted to light sources at the outside of buildings and installations in the Park were also occasionally recorded. At the lights, only manual recording and sampling was performed and no automated traps were employed.

**Soil sieving**

To improve the chances of finding small terrestrial molluscs, samples of leaf litter and upper soil were dried and finely sieved. They were then carefully searched under a stereomicroscope, while live specimens and empty shells were collected using forceps.

**Mist nets and harp trap**

Bats were captured with two differents methods: (1) monofilament mist-nets (Ecotone, Poland) and (2) a harp trap. Mist-nets consisted of a fine nylon mesh separated into 3-5 shelves. Net length (8 m, 12 m, 16 m or 20 m) was chosen according to the width of the sampling passage. The harp trap consisted of four dismountable metal frames (2.0 m [height] x 1.5 m [width]) separated from each other by 15 cm. Each frame had vertical lines of thin wires of monofilament fishing lines, fastened 2.5 cm apart. A collecting bag prevented bats from flying or crawling out. After capture, two wing punches were collected per individual and preserved in 90% ethanol in 1.5 ml tubes.

#### Aquatic fauna

Fishes and decapod crustaceans were generally collected using hand nets (dip nets of varying mesh size) and (fishes only) by electrofishing. Crabs were also collected by hand. Fishes were fixated in formalin (10% solution) for two weeks and then transferred to 70% ethanol for long-term preservation and collection storage. Prior to fixation in formalin, tissue clips (muscle or fin) were taken for DNA analyses and preserved in 99.6% ethanol. Crustaceans were fixed in 96% ethanol and later transferred to 70% ethanol.

#### Terrestrial flora and funga (including lichens)

Vascular plants, including underground parts, were collected (for seed plants in flowering or fruiting stage). Epiphytic plants and flowering or fruiting twigs or branches of trees were cut using telescope scissors, which enabled sampling up to 6 m above ground level. Fresh plant material was pressed between newspaper and cardboard and dried on a gas heater for 24-48 h. The drying process was regularly monitored until dessication was completed. Leaf tissue from each herbarium specimen was separately dried in tea-filter bags with silica gel. Epiphytic, epilithic and terrestrial bryophytes, fungi and lichens were removed from their substrate and air-dried in paper bags.

#### Aquatic microalgae (diatoms)

For sampling of aquatic diatoms from running waters, the organic layers covering submerged stones were removed with a fresh toothbrush and transferred with some water to microtubes. Half of the sample was conserved in 97% ethanol (final concentration approximately 70%) for eDNA metabarcoding (Zimmermann et al. 2015), as well as for classical identification by light (LM) and scanning electron microscopy (SEM) (Mora et al. 2019). The other half was kept alive for establishing cultures (Skibbe et al. 2018).

### Data recording

**MyFieldBook app**

For a fast and comprehensive digital data capture in the field, smartphones are appropriate multi-functional devices and readily available. In order to provide a tool fulfilling generic requirements for biodiversity data recording, a prototypic mobile app was developed for the rapid data capture of all sorts of samples and observations in the field.

**Figure 4.**
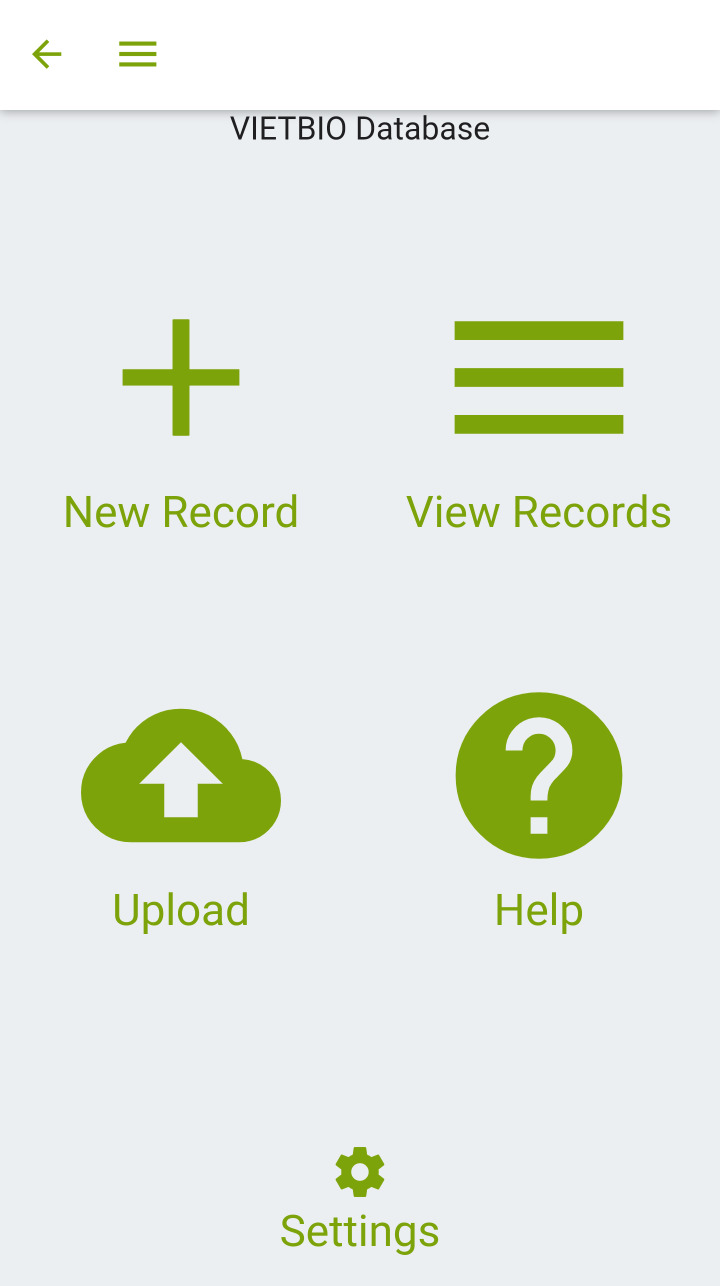
Digital data entry of samples and observations with MyFieldBook app.

**Figure 5.**
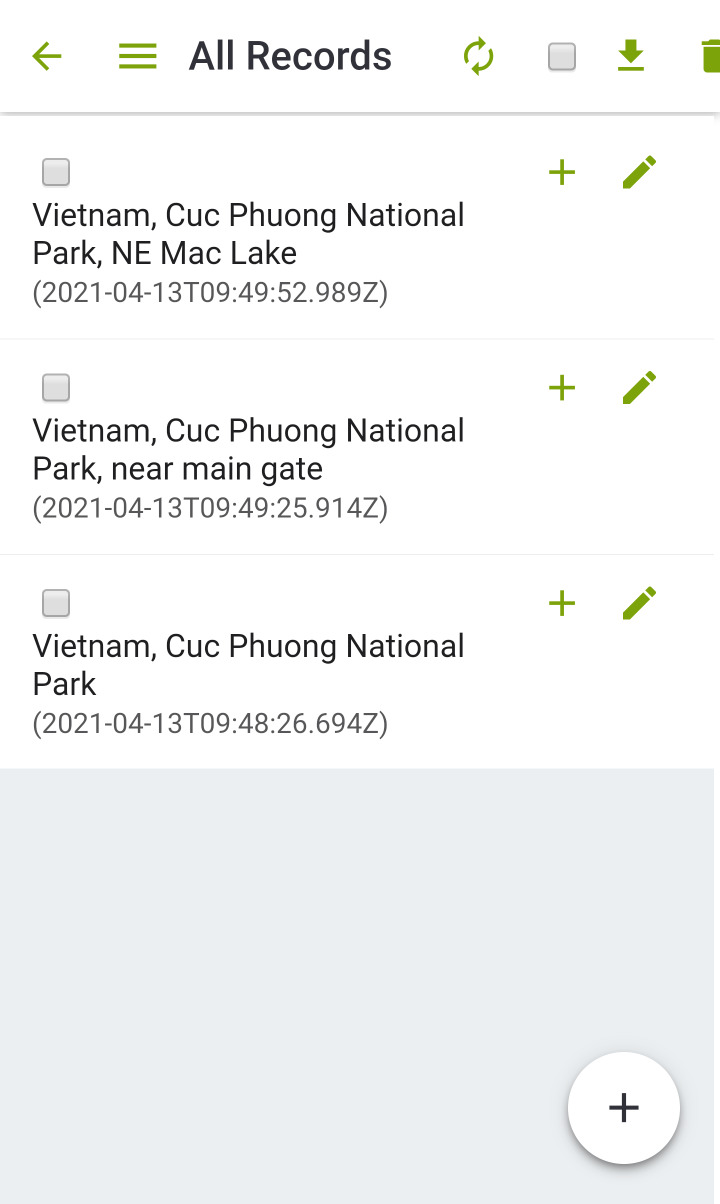
The app MyFieldBook allows a complete digital workflow from the field to the lab by using QR-codes as unique identifiers.

**Figure 6.**
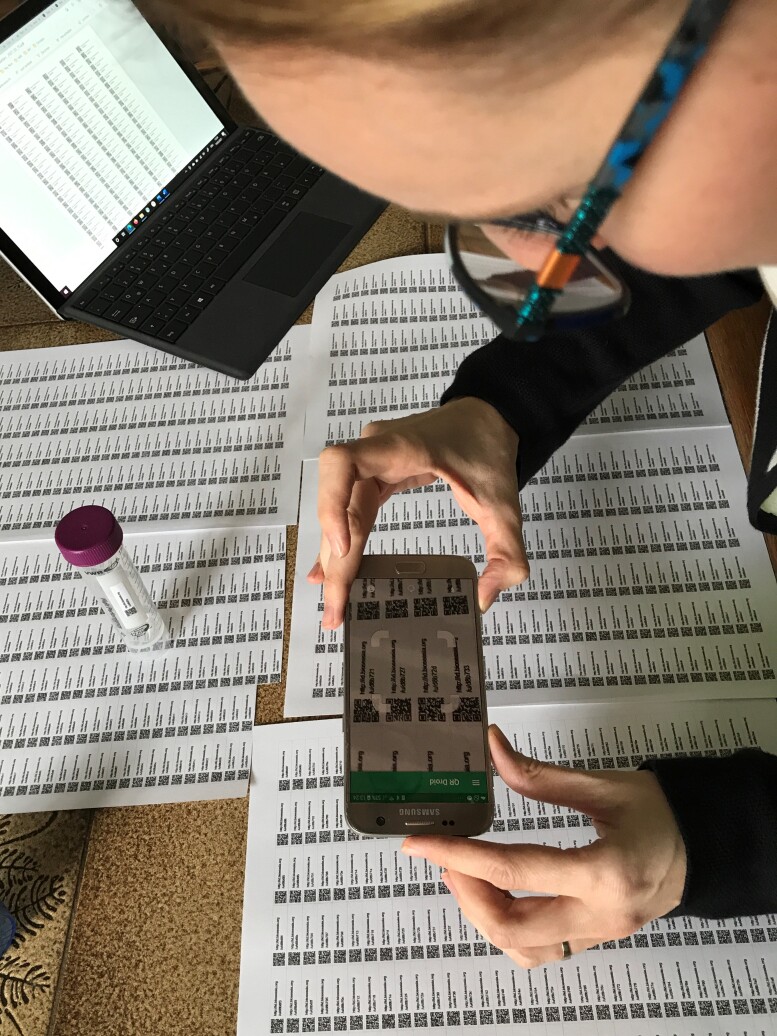
Scanning of QR codes by using the MyFieldBook app.

This app (MyFieldBook; https://myfieldbook.online/) is primarily designed to allow for easy initial (meta-)data capture and to help register all records and samples from their origin in the field via unique identifiers. The assignment of unique IDs is implemented via scanning QR codes directly in the field, which are associated with each and every type of record. In addition, the app allows for fast-track capturing of standard meta-data (date, time, coordinates) for each record, which can be enriched with more information (e.g. scientific name, sequence data etc.) later on or in the lab. This allows for a complete digital workflow from the field to the lab/collection by using QR-codes as unique persistent identifiers (Figs 4, 5). Participants of the field trip tested the app with different devices under field conditions, provided feedback and reported errors and additional requirements (Fig. 6) and by this, facilitated the development of the app into a generic tool as a digital alternative for any traditional field book.

Its main functionality comprises:

- Data entry for most common biodiversity occurrence information: What was when and where found or observed?
- Image capture for samples, habitats and additional documentation
- Retrieval of coordinates via GPS
- QR code and barcode scanning for sample management in the field
- Multiple samples per biodiversity occurrence record
- Customisable data model, so users can add additional fields they need
- Multiple databases per device, so different projects with different data models can be accommodated
- Offline data capture (no network connection needed in the field)
- SQLite and JSON data export
- Data upload to server-sided databases (for compiling data from different devices and different users in one database)
- soon freely available for Android and iOS

Due to differently established workflows for the botany team, the app was tested and applied in the project only for zoological records in Cuc Phuong National Park. Specifically, it was used for primary field data recording including image capture with QR code scanning for subsequent data management and data upload for further processing.

### Identification

Specimens were identified according to standard reference works and validation was performed by specialists of the respective taxa. Where data availability allowed, morphological identification was additionally confirmed via DNA barcoding (e.g. for decapods, Hymenoptera and fungi). Geographic coordinates were primarily recorded using GPS via the app on smartphones and later mapped and checked using Google Maps or Open Street Map.

## Results

The aim was to identify all specimens at least to family level and, depending on the availability of identification keys and expertise, to genus or species level. The records and samples of Animalia (Chordata, Arthropoda, Mollusca) included at least 18 orders and 81 families. The samples of vascular plants included 79 families and at least 175 genera and 229 species, those of bryophytes representing 12 families, 18 genera and at least 22 species. Based on 210 established diatom cultures, 110 species in 40 genera from 16 families and eight orders were recorded; for the seven mixed samples of the sampling campaign of 2019, we expect at least a doubling of the species numbers. In addition to the high number of species, the diatom flora of Vietnam seems to be quite different from the well-known European diatom flora (Hofmann et al. 2013): for 10 strains, we were uncertain about the correct genus name because neither available molecular nor morphological data were congruent and only 21 of the strains were identified to the species level by using fine grained integrative taxonomic concepts. A just-published Russian book on the diatom flora of Southeast Asia (Glushchenko et al. 2021) needs to be consulted and their species concepts compared. The fungal collections encompassed 42 macrofungi and 54 lichens. The ten macrofungi, subjected to DNA barcoding, resulted in nine species representing eight genera, three families, three orders, two classes and two phyla, with a predominance of the order Polyporales. The lichen fungi were more phylogenetically diverse, representing 40 species, 23 genera, 13 families, ten orders, four classes and one phylum, mostly representing the class Lecanoromycetes.

This editorial paper is followed by individual data papers, which will include more detailed information on the findings of Lepidoptera and Trichoptera; Diptera (incl. bat flies) and Hymenoptera; Coleoptera, Odonata and Neuroptera (Mantispidae); Crustacea (Decapoda) and fishes; Mollusca; Amphibia and reptiles; Chiroptera; vascular plants and bryophytes; diatoms; macrofungi and lichens.
